# Peritoneal Filariasis in the Horse

**Published:** 1903-04

**Authors:** John J. Repp

**Affiliations:** Ames, Iowa


### PERITONEAL FILARIASIS IN THE HORSE.

By John J. Repp, V.M.D.,
AMES, IOWA.

I have frequently observed in the peritoneal cavity of horses in Iowa a nematode worm about as thick as a knitting-needle, and two to six inches in length, with the tail disposed in a spiral manner. My observations have extended over horses varying in age from one to thirteen years. The worms have never in my experience been in great numbers, such as described by MacGillivray, nor have I ever been able to trace evil effects to them. Recently I have identified this worm as the *Filaria equina* Abildgaard (*Filaria papillosa* Rudolphi). Neuman gives the following description:

Body long, filiform, white, attenuated at both ends, and especially behind; mouth small and provided with a chitinous infundibuliform ring, the border of which is divided into four rounded and salient papillæ. Outside this ring are other four submedian papillæ in the form of spinules. *Male*, 6 cm. to 8 cm. long; tail curved in a spiral manner and having eight papillæ on each side, of which there are four pre-anal and four post-anal; two unequal spiculæ enveloped in a transparent sheath. *Female*, 9 cm. to 15 cm. long; tail slightly spiral and terminated in a papilla preceded by two others; vulva situated near the anterior extremity. Ovoviviparous.

These worms frequently occupy the tunica vaginalis along with the testicle, and come out through the incision when castration is being performed. Their presence is recorded in the pleural sac, arachnoid space, subperitoneal connective tissue, and in the substance of the diaphragm, but I have never met with them in any of these positions.

